# Assessment of associated credit risk in the supply chain based on trade credit risk contagion

**DOI:** 10.1371/journal.pone.0281616

**Published:** 2023-02-16

**Authors:** Xiaofeng Xie, Fengying Zhang, Li Liu, Yang Yang, Xiuying Hu

**Affiliations:** 1 Innovation Center of Nursing Research, Nursing Key Laboratory of Sichuan Province, West China Hospital, West China School of Nursing, Sichuan University, Chengdu, Sichuan, China; 2 West China School of Nursing, West China Hospital, Sichuan University, Chengdu, Sichuan, China; 3 School of Economics Mathematics, Southwestern University of Finance and Economics, Sichuan, China; URV: Universitat Rovira i Virgili, SPAIN

## Abstract

Assessment of associated credit risk in the supply chain is a challenge in current credit risk management practices. This paper proposes a new approach for assessing associated credit risk in the supply chain based on graph theory and fuzzy preference theory. First, we classified the credit risk of firms in the supply chain into two types, namely firms’ “own credit risk” and “credit risk contagion”; second, we designed a system of indicators for assessing the credit risks of firms in the supply chain and used fuzzy preference relations to obtain the fuzzy comparison judgment matrix of credit risk assessment indicators, on which basis we constructed the basic model for assessing the own credit risk of firms in the supply chain; third, we established a derivative model for assessing credit risk contagion. On this basis, we carried out a comprehensive assessment of the credit risk of firms in the supply chain by combining the two assessment results, revealing the contagion effect of associated credit risk in the supply chain based on trade credit risk contagion (TCRC). The case study shows that the credit risk assessment method proposed in this paper enables banks to accurately identify the credit risk status of firms in the supply chain, which helps curb the accumulation and outbreak of systemic financial risks.

## 1. Introduction

As economic globalization progresses, supply chains are becoming more and more complex, encompassing various industries and regions as well as multiple members and relationships. At present, a supply chain has evolved into a complex system composed of multiple interconnected firms [1]. The interrelationships among firms in the supply chain have led to credit contagions from one firm to another and have formed the contagion network of the associated credit risk in the supply chain [2]. The associated credit risk in the supply chain presents a strong contagion effect, which directly endangers banks that provide loans to firms in the supply chain [3]. Therefore, when banks assess the credit risks of firms in the supply chain, they must focus on assessing the contagion effect of associated credit risk in the supply chain.

Examples of the contagion effect of credit risk in the supply chain abound. For instance, the Reward Group, a diversified firm integrating the three major industries of commodities, dairy and tourism real estate in China, filed for bankruptcy and reorganization in 2019 with a debt of RMB 6.094 billion. Due to its default on the accounts payable to its main upstream and downstream firms Tianjin Golden Eagle Trading and Kyushu Eagle, the two companies went into financial crisis soon after the bankruptcy of the Reward Group [4]. For another example, the investors in the collateralized debt obligations suffered a large, unexpected loss in May 2005 due to the credit risk diffusion when Ford and General Motors were downgraded to junk status [5].

Under banks’ traditional credit risk assessment framework, it is generally believed that the credit status of a firm changes independently [6]. Therefore, when traditional credit risk assessment methods are used to assess firms in the supply chain, they are often isolated from the supply chain environment and only assess the credit status of a single firm without considering the contagion of credit risk among firms in the supply chain, resulting in inaccurate assessment results [7, 8]. Therefore, it is difficult to provide scientific theoretical bases for banks to make credit decisions and control risks [9, 10].

As a short-term financing strategy widely adopted by firms in different countries such as China, the U.S. and the U.K., trade credit has gradually become a common practice among firms in the supply chain [11]. About 70% of American businesses and 80% of British businesses extend trade credit to customers. As China’s financial system is not perfect, the impact of trade credit on the development of firms is even greater than that of bank credit [12, 13]. However, the widespread use of trade credit has resulted in an endless stream of credit crises [14]. For example, in 2008, the bankruptcy and reorganization of General Motors led to its inability to honor its trade credit obligations, which caused many auto parts suppliers in China, Japan, South Korea and other countries to face huge financial difficulties and operational crises. In 2015, V&D, a well-known department store chain in the Netherlands, went bankrupt, putting hundreds of suppliers at risk or into bankruptcy. The crisis involved several associated companies in the supply chain network and affected several banks and financial institutions. It brought huge risks and hidden dangers to the stability of the financial system. Fewings believes that trade credit leads to the formation of a Markov chain among firms, and that the credit crisis of any firm in the chain may lead to the break of the whole chain, which will bring negative effects to banks and affect the volatility of the financial market [15].

Based on this, this paper classifies credit risk of firms in the supply chain risk contagion network into two types, namely firms’ “own credit risk” and contagion risk caused by other firms in the supply chain with which firms have commercial credit relationships, or in other words, “trade credit risk contagion (TCRC)”. Firms’ “own credit risk” in the supply chain refers to firms’ internal negative factors (such as organizational structure, imperfect management system or deficient product process or production technology) or the default risk triggered by fluctuations in the external macroeconomic environment. TCRC in the supply chain risk contagion network refers to the phenomenon where one firm in the supply chain risk contagion network defaults, causing other firms in the network to default or suffer from a higher probability of default through trade credit risk contagion channels. In the supply chain risk contagion network, firms’ “own credit risk” is indicated as nodes whereas TCRC among firms directed edges.

This paper puts forward a new method to assess the associated credit risk in the supply chain in view of the contagion effect of credit risk that is widespread among firms in the supply chain. Since trade credit is the most widely used financing strategy between upstream and downstream firms in the supply chain, credit risk contagion for many firms mainly comes from trade credit among firms in the supply chain. In other words, trade credit among firms in the supply chain is the source of contagion effect of the associated credit risk in the supply chain. The present study is conducted from the perspectives of graph theory and fuzzy preference theory. It constructs a credit risk assessment model based on TCRC and assesses the credit risk of firms in the supply chain and reveals the contagion effect of associated credit risk in the supply chain.

At present, the perspective of supply chain enterprise credit evaluation is relatively limited, and there is still little research that includes the correlation between enterprises and embeds the contagion effect of credit risk between enterprises. Su et al. enriched the credit risk characteristic system of enterprises from external banks and logistics enterprises but did not consider the transaction behavior between supply chain enterprises and their upstream and downstream enterprises [16]. Zhu et al. studied the credit risk of small- and medium-sized enterprises from the perspective of the supply chain but excluded transaction-related indicators such as trade credit [17]. Martin et al. considered the relationship between upstream and downstream enterprises and core enterprises as the main factor affecting the credit risk of supply chain enterprises [2]. However, they did not consider the risk contagion of counterparty enterprises [2]. Relevant studies have not evaluated the credit risk assessment of enterprises in the supply chain from the perspective of credit risk contagion [6–8], so the evaluation model constructed is not suitable for the evaluation of supply chain enterprises’ credit risk. This study examines not only the credit risk of each nodal enterprise but also the direct and indirect contagion effect between nodal enterprises’ credit risks. Therefore, under the supply chain network structure, the related credit risk assessment caused by business credit studied in this paper has important theoretical value and practical significance.

The remainder of this paper is structured as follows: Section 2 provides the associated credit risk assessment model based on TCRC, and Sec. 3 introduces the basic model for assessing firms’ own credit risk. Section 4 presents the derivative model for assessing TCRC. Section 5 presents an analysis of the framework through case simulation. Section 6 concludes and provides further discussion.

## 2. Setup of the model for assessing associated credit risk based on TCRC

Consider a network composed of firms *S*_1_, *S*_2_,⋯,*S*_*n*_ in the supply chain. Node *i* stands for firm *S*_*i*_, and the direction of edges (*i*,*j*) is *i*→*j*, which means trade credit flows from *S*_*j*_ to *S*_*i*_. If *S*_*j*_ extends trade credit to *S*_*i*_, once *S*_*i*_ goes into a financial crisis, its credit risk will spread to *S*_*j*_. So, (*i*,*j*) represents the pathway of credit risk contagion between *S*_*i*_ and *S*_*j*_. The weights of (*i*,*j*) are (*I*,*t*), of which *I* stands for the line of trade credit extended to *S*_*i*_ by *S*_*j*_, and *t* represents the duration of trade credit. Assume that credit risk contagion among firms can work not only directly but also indirectly. Besides, the number of firms and the trade credit contracts (*I*,*t*) among firms remain unchanged in the analysis period. Therefore, there is neither newly added nor removed credit risk.

Firms’ credit risk in the supply chain risk contagion network is indicated as

$$TR=(R_{1},\;R_{2},\;\cdots ,\;R_{n})=R_{s}+R_{c},$$ (1)

where *R*_*s*_ = (*R*_*s*1_, *R*_*s*2_,⋯,*R*_*sn*_), representing firms’ own credit risk, and *R*_*c*_ = (*R*_*c*1_, *R*_*c*2_, ⋯, *R*_*cn*_) representing firms’ TCRC; *R*_*i*_ (*i* = 1, 2, ⋯, *n*) represents the credit risk of the *i*th firm; *R*_*si*_ stands for the own credit risk of the *i*th firm; and *R*_*ci*_ stands for the credit risk contagion of the *i*th firm.

To measure *R*_*s*_, which stands for firms’ “own credit risk”, this paper designs a system of indicators for assessing firms’ own credit risk in a supply chain scenario, focusing on firms’ credit history, relationships with core firms and the overall operation of the supply chain. Both quantitative and qualitative indicators are included in the indicators for assessing firms’ own credit risk. In this paper, we normalized the quantitative indicators to eliminate the inconsistency of assessment results and then established a Fuzzy Comparative Judgment Matrix (FCJM) for the quantitative indicators. As for qualitative indicators, due to the limitations of decision makers, it is often difficult to fully understand the nature of the object of assessment. Use of fuzzy preference relations to construct an FCJM enables scientific measurement of qualitative indicators and maximizes the integration of various types of information that can help judge the credit risk of firms in the supply chain.

*R*_*c*_, which refers to firms’ “TCRC” in the supply chain risk contagion network, is related to such factors as the structure of the risk contagion network constituted by trade credit in the supply chain, the source of contagion and the size of the own credit risk of the firms that fall victim to credit contagion. Assume that *A* = (*a*_*ij*_)_*N*×*N*_ is a matrix of risk values that represents the paths and degree of credit risk contagion among firms in the supply chain risk contagion network. If there is an edge pointing from *i* to *j*, then *a*_*ij*_ = *I*^*t*^, where *I*^*t*^∈(0,1] reflects the extent to which firm *S*_*j*_ in the chain is affected by the credit risk of *S*_*i*_. If *a*_*ij*_ = 0.45, it means that *S*_*i*_’s own credit risk may be contagious to *S*_*j*_ with a probability of 45%.

*R*_*c*_, which refers to firms’ “TCRC” in the supply chain risk contagion network, is indicated as

$$R_{c}=\sum_{i=1}^{m}A^{i}R_{s},$$ (2)

where the reachability matrix $\sum_{i=1}^{m}A^{i}$ measures the degree of contagion of associated credit risk in the supply chain risk contagion network [18, 19]; *i* refers to the length of the pathway and represents the credit risk contagion distance, which can be obtained by calculating the number of associated paths between any two enterprises in the supply chain; and *m* stands for the maximum contagion distance in the supply chain risk contagion network and represents the maximum value in the path length set between enterprises.

We substitute Eq (2) into Eq (1) to obtain the credit risk of firms in the supply chain risk contagion network: $TR{=R}_{s}+R_{c}=(E+\sum_{i=1}^{m}A^{i})R_{s}$. In particular, if *m*→+∞, and we multiply both sides of Eq (3) by Matrix (*E*−*A*), then $(E-A)TR=(E-A^{m+1})R_{s},\;and\;TR={\left(E-A\right)}^{-1}(E-A^{m+1})R_{s}={\left(E-A\right)}^{-1}\left(E-{\underset{m\to +\infty }{lim}A}^{m+1}\right)R_{s}$. In fact, it is rare to see all firms in the supply chain risk contagion network forming circles due to trade credit. Therefore, ${\underset{m\to +\infty }{lim}A}^{m+1}=0$, and *TR* = (*E*−*A*)^−1^*R*_*s*_.

In summary, the TCRC-based model for assessing credit risk of firms in the supply chain is

$$TR=R_{s}+R_{c}=\left\{ \begin{array}{c} (E+\sum_{i=1}^{m}A^{i})R_{s},\;if\;m\;\mathrm{is}\;a\;\mathrm{limited}\;\mathrm{positive}\;\mathrm{integer}\\ {\left(E-A\right)}^{-1}R_{s},\;if\;m\to +\infty \end{array}\right..$$ (3)

## 3 The basic model for assessing firms’ own credit risk

**Fig 1:**
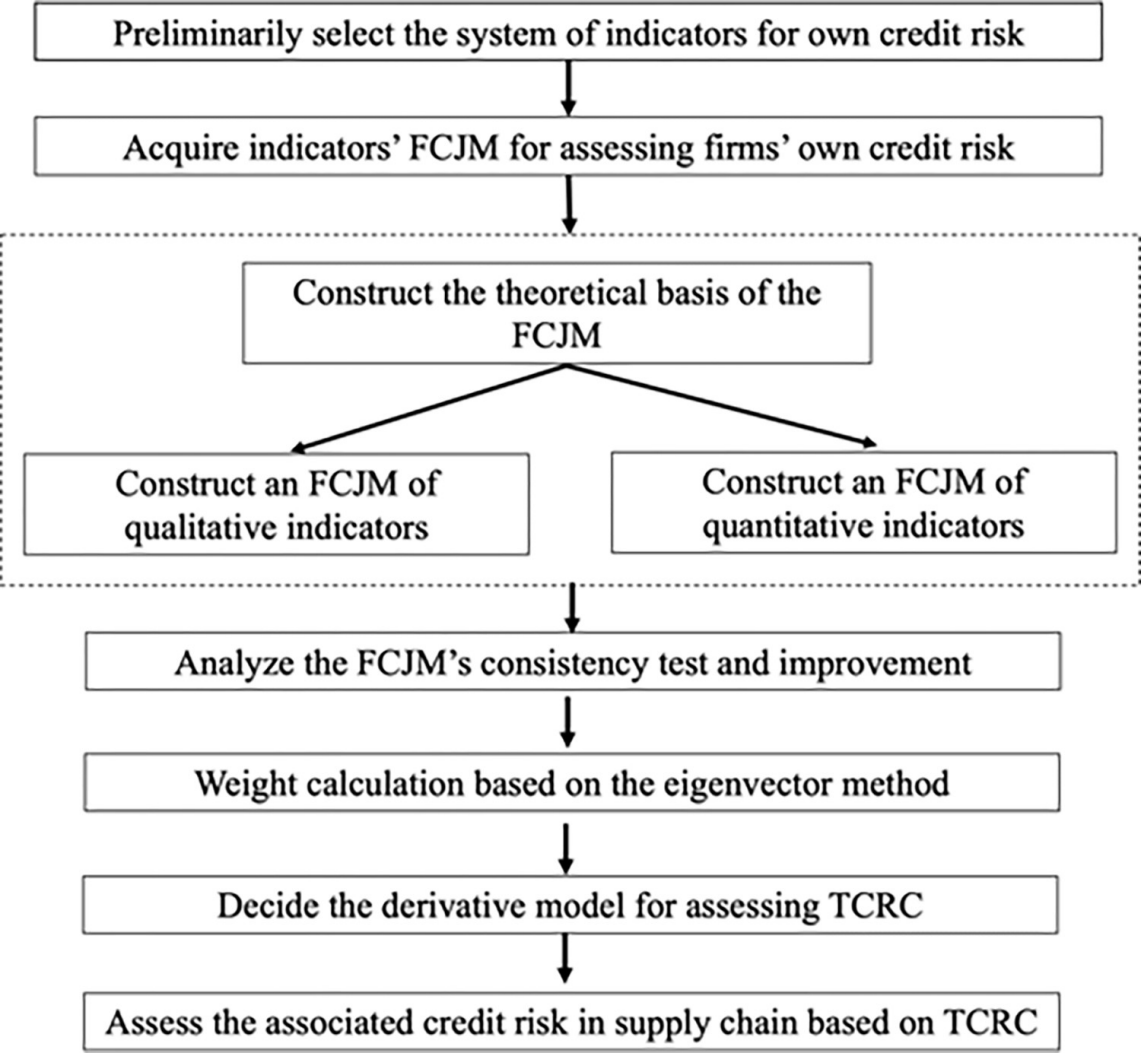
The visual process to evaluate the credit risk of enterprises in the supply chain.

Drawing from the system of indicators for assessing firms’ credit risk used by banks and based on characteristics such as the operation of firms and close connections among firms in the supply chain, we constructed a system of indicators for assessing firms’ own credit risk with characteristics of the supply chain; besides, we quantified the indicators for assessing firms’ own qualitative credit risks using fuzzy preference relations and obtained an FCJM of the indicators for assessing firms’ own credit risk; finally, after passing the consistency check, we obtained the weight vectors of the system of indicators for assessing firms’ own credit risk using the eigenvector method and then carried out an assessment of the own credit risk of firms in the supply chain risk contagion network. The visual process for financial institutions to evaluate the credit risk of enterprises in the supply chain is shown in Fig 1.

### 3.1 The system of indicators for assessing firms’ own credit risk

Supply chain finance has been more and more widely used in supply chain enterprises. At present, Bank of America, Deutsche Bank, HSBC, Standard Chartered, and Citibank are also developing "supply chain finance" services, according to the Aberdeen Group report. In the past, commercial banks paid more attention to the investigation of financial indicators of SMEs in addition to their credit history and mortgage value before providing loans to SMEs [20]. These indicators include profitability, operating ability, solvency, and so on [21, 22]. However, some more prudent banks will add necessary qualitative indicators. For example, when China Construction Bank (CCB) evaluates the credit quality of the enterprises, it not only considers the above financial indicators but also integrates the soft information such as the economic development level of the enterprises’ region and the policy environment of the enterprises’ credit period [23]. At the observer level, CCB pays more attention to the solvency of the enterprises, focusing on evaluating the short-term and long-term solvency of the enterprises and examining whether the enterprises have default records in the bank or other banks in the recent three years. Based on this, the index system of this paper is consistent with financial practice and literature research. When the banks evaluate the credit risk of enterprises in the supply chain, both quantitative indicators such as financial indicators and qualitative indicators such as macroeconomic environment, policy environment, and credit history are considered. Thus, the credit risk evaluation model constructed in this section is a hybrid model combining qualitative and quantitative indicators [20–23].

For an enterprise in the supply chain, to evaluate its credit risk, it should not only examine its financial indicators, but also examine its counterparty credit, the stability of the relationship with the core enterprise, the control ability of the transaction process, the enterprise’s past transaction records and the operation status of the entire supply chain. Therefore, the credit risk evaluation mechanism of enterprises under the supply chain scenario has undergone fundamental changes. To establish the credit risk evaluation index system of supply chain enterprises, it should follow the principles of comprehensiveness, scientificity, pertinence, fairness, legality, and operability. This paper draws on the basic framework of traditional business credit evaluation and carries out the design according to the characteristics of supply chain enterprises themselves [20–23], that is, combining the characteristics of supply chain and the credit level of borrowers, focusing on the solvency and credit status of enterprises and counterparties.

Drawing on the research carried out by Xiong et al. [20], Hu et al. [21] and Dai [22], this paper constructs the system of indicators for assessing firms’ own credit risk in the context of a supply chain (see Table 1) by selecting from four aspects, namely external environment of the supply chain, relationship status in the supply chain, own factors of firms in the supply chain and factors of associated firms in the supply chain. The system of indicators for assessing firms’ own credit risk consists of 4 first level indicators and 31 second level indicators.

**Table 1:** The system of indicators for assessing the own credit risk of firms in the supply chain.

First-level indicators	Second-level indicators	Third-level indicators	Indicator description
External environment of the supply chain	Macro-environment	Macroeconomic environment *X*_1_	The cycle of economic development, GDP growth trend
	Industry prospects	Legal and political environment *X*_2_	Relevant preferential policies and laws and regulations of the state
		Industry growth index *X*_3_	Whether the industry is emerging, mature or declining
		Level of industry competition *X*_4_	The intensity of industry competition
Relationship status in the supply chain	Relationship tightness	Relationship contract strength *X*_5_	Whether the two parties have signed a long-term supply and marketing contract
		Commitment *X*_6_	Willingness of core firms in the supply chain to maintain long-term relationships with the firm
	Relationship durability	Cooperation time *X*_7_	Time of cooperation between core firms in the supply chain and the firm
	Relationship closeness	Managers’ personal relationships *X*_8_	The state of personal relationships between key managers of core firms in the supply chain and those of the firm
		Employees’ personal relationships *X*_9_	The state of personal relationships between employees of core firms in the supply chain and those of the firm
	Performance status	The default rate *X*_10_	Number of historical defaults/total transactions in the supply chain
	Position of the enterprise in the supply chain	Product price advantage *X*_11_	The relationship between product price and market average price level
		The product can be replaced by *X*_12_	The degree of homogeneity of enterprise products and similar products on the market
Own factors of firms in the supply chain	Firm quality	Firm management status *X*_13_	Firm management situation and leadership structure
		Employee’quality *X*_14_	Level of education, mastery of technology
		Quality of financial disclosure *X*_15_	Review of financial statements and information disclosure
	Profitability	Return on equity *X*_16_	Total net profits/Average total net assets
		Return on sales*X*_17_	Sales profit/Sales revenue
	Operating capacity	Inventory turnover *X*_18_	Cost of sales/Average inventory
		Sales growth rate *X*_19_	Sales revenue growth in the current period/Sales revenue of the previous period
	Solvency	Current ratio *X*_20_	Current assets/Current liabilities
		Quick ratio *X*_21_	(Current assets—Inventory)/Current liabilities
		Asset-liability ratio *X*_22_	Total liabilities/Total assets
		Interest rate coverage multiple *X*_23_	Earnings before interest and tax /Interest expense
	Development potential	Sales revenue growth rate *X*_24_	Proportion of the difference between the current sales profits and the sales profits of the prior period
		Net profit growth rate *X*_25_	(Net profit for the period—Net profit for the corresponding period of the previous year)/Net profit for the same period of the previous year
		Total assets growth rate *X*_26_	(Total assets for the period—total assets for the same period of the previous year)/total assets for the same period of the previous year
	Credit history	Contract performance history *X*_27_	Whether there has been a breach of contract
Factors of associated firms in the supply chain	Credit ratings of associated firms	Credit rating *X*^28^	Credit ratings of associated firms in the supply chain at banks
	Industry characteristics of associated firms	Industry status *X*_29_	The industry status of associated firms in the supply chain
	Profitability of associated firms	Return on sales *X*_30_	Sales profit/Sales revenue
	Solvency of associated firms	Quick ratio *X*_31_	(Current assets -Inventory)/Current liabilities

### (1) External environment of the supply chain

The risk of default of firms in the supply chain caused by fluctuations in the external environment of the supply chain is known as systemic risk for firms. The external environment of the supply chain includes dimensions such as macroeconomic environment, political environment, industry prospects and level of industry competition.

### (2) Relationship status in the supply chain

Relationship status in the supply chain includes the tightness, durability, and closeness of relationships among firms in the supply chain, history of supply chain performance and status of firms in the supply chain. When granting credit to firms in the supply chain, banks usually assess their solvency from not only their own credit status, but also the perspective of the whole supply chain, specifically the status of their relationships with associated firms, history of their contract performance in the supply chain and their status in the supply chain. This enables banks to assess risk more accurately and reduces the risk of adverse selection of firms to a certain extent.

### (3) Own factors of firms in the supply chain

Banks focus on assessing such aspects as the operation and management status as well as solvency of firms in the supply chain as borrowers. The own factors of firms in the supply chain include their quality, profitability, operating capacity, solvency, development capacity and credit history.

### (4) Factors of associated firms in the supply chain

The indicators in this aspect mainly include the credit ratings, industry characteristics, operating capacity, and solvency of associated firms in the supply chain. Examination of the qualifications of associated firms in the supply chain is mainly aimed at finding out whether the core supply chain enterprises have the ability and credit to repay the debts. In supply chain finance, the core enterprise in the supply chain, as the core of the transaction flow, information flow and capital flow in the whole supply chain, can play a counter-guarantee role in the financing of the SMEs. The business prospect and credit status of core enterprises are directly related to the quality of their transactions with the SMEs. If the core enterprise has good business conditions and good credit quality, it means that the core enterprise has good debt paying ability and willingness, which can reduce the credit risk faced by the bank to a considerable extent.

**Fig 2:**
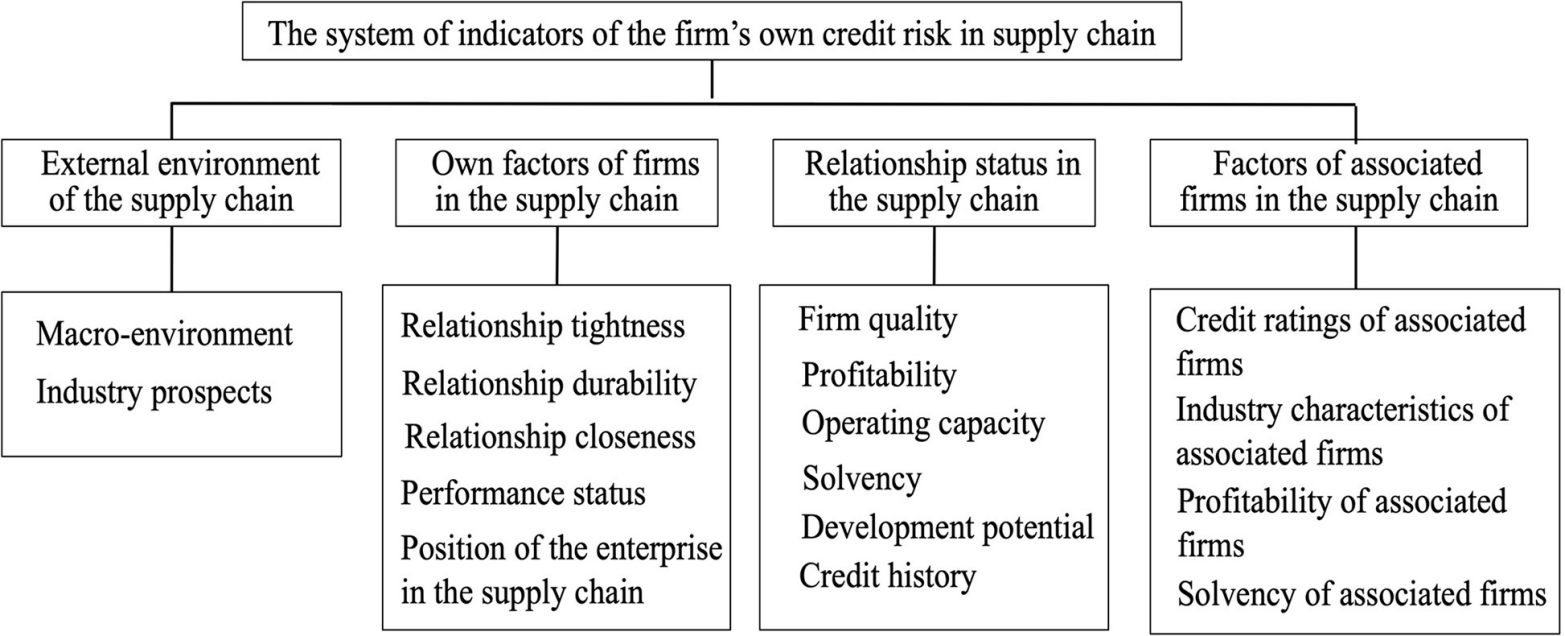
The system of indicators of firm’s own credit risk.

The system of indicators of the firm’s own credit risk in the supply chain is shown in Fig 2. The indicators system contains the specific own credit risk evaluation index of the enterprises in the supply chain.

### 3.2 FCJM of the indicators for assessing firms’ own credit risk

#### 3.2.1 Theoretical basis of FCJM

In the process of risk assessment, if the amount of data available for the relevant assessment indicators is small and the results of risk assessment also depend on the experience, ability, and personal preference of the decision makers, then it is necessary to establish an assessment system with both “quantitative and qualitative” indicators. In addition, the assessment results are characterized by a variety of forms and vague descriptions subject to the different types and nature of the assessment indicators as well as the assessors’ own cognitive competence and lack of information. In recent years, with the widespread application of fuzzy preference theory in the field of economic decision making, many scholars at home and abroad have used fuzzy preferences to make up for this deficiency. The commonly used fuzzy preferences include classical fuzzy preference, hesitant fuzzy preference, and interval fuzzy preference. The use of fuzzy preference theory for risk decision making refers to the decision makers’ use of certain fuzzy evaluation criteria to obtain an FCJM by comparing every two objects of assessment before using the FCJM to assess the risk of each object of assessment. Prediction of the future risk of each object of assessment can provide a basis for decision makers to formulate risk management measures.

Fuzzy preference is a judgment method based on a scale of 0.1–0.9. The scale of 0.1–0.9 is uniformly distributed on the interval [0,1] and symmetric on both sides centered at 0.5. Orlovsky [24] defines the FCJM on a decision set *X* based on fuzzy preferences on a scale of 0.1–0.9 as

$$R={\left(r_{ij}\right)}_{n\times n}\subseteq X\times X,$$ (4)

where *r*_*ij*_+*r*_*ji*_ = 1, and *r*_*ii*_ = 0.5; *r*_*ij*_ = *μ*_*R*_(*X*_*i*_, *X*_*j*_), where *μ*_*R*_: *X*×*X*→[0,1] is the membership function on the decision set *X*. *r*_*ij*_∈[0,1] represents the result of comparing the importance of the assessment object *X*_*i*_ with *X*_*j*_. In other words, *r*_*ij*_ measures the extent to which the decision maker considers the assessment object *X*_*i*_ to be more important than *X*_*j*_.

In particular, *r*_*ij*_ = 0.5 means that the assessment object *X*_*i*_ is equally important as *X*_*j*_. In other words, the decision maker has no preference between *X*_*i*_ and *X*_*j*_, which can be expressed as *X*_*i*_~*X*_*j*_; *r*_*ij*_ = 1 means that *X*_*i*_ is extremely more important than *X*_*j*_, or in other words, the decision maker has an extreme preference for *X*_*i*_ when comparing it with *X*_*j*_, which can be expressed as *X*_*i*_≻≻*X*_*j*_. *r*_*ij*_>0.5 means that *X*_*i*_ is more important than *X*_*j*_, or in other words, the decision maker prefers *X*_*i*_ when comparing it with *X*_*j*_, which can be expressed as *X*_*i*_≻*X*_*j*_. A more detailed interpretation of the assessment results is shown in Table 2 below.

**Table 2:** Connotations of the fuzzy judgment of credit risk based on a scale of 0.1–0.9.

Judgment scale	Connotation
0.5	The two objects of assessment have the same amount of credit risk.
0.6	One assessment target has slightly higher credit risk than the other.
0.7	One object of assessment has obviously higher credit risk than the other.
0.8	One object of assessment has strongly higher credit risk than the other.
0.9	One object of assessment has extremely higher credit risk than the other.
Other values between 0 and 1	If the result of comparing assessment object *i* with *j* is *r*_*ij*_, then the result of comparing assessment object *j with i* is: *r*_*ji*_ = 1−*r*_*ij*_.

#### 3.2.2 Construction of an FCJM of qualitative indicators

Decision makers mainly assess qualitative indicators based on subjective judgments and objective information about the indicators, and it is often difficult to measure the results with precise values. As a result, the assessment results based on qualitative indicators are generally characterized by vague expressions and strong randomness of judgments. Therefore, this paper uses random judgment to express decision makers’ assessment opinions on qualitative indicators.

Based on the previous analysis, the FCJM of firms *S*_1_, *S*_2_,⋯,*S*_*n*_ in the supply chain risk contagion network is defined under a certain qualitative indicator *X*_*l*_ on a scale of 0.1–0.9 as

$$R^{\left(l\right)}={\left(r_{ij}^{\left(l\right)}\right)}_{n\times n},$$ (5)

where $r_{ij}^{\left(l\right)}$ is a random variable whose probability density function is ${f(r}_{ij}^{\left(l\right)})$, and cumulative probability distribution function is ${F(r}_{ij}^{\left(l\right)})$. $r_{ij}^{\left(l\right)}$ measures the degree of the decision makers’ preference for the size of the own credit risk of firms *S*_*i*_ and *S*_*j*_ in the supply chain risk contagion network under the qualitative indicator *X*_*l*_.

In reality, decision makers tend to hesitate between certain assessment results, which is mainly characterized by hesitant fuzzy preference. Based on this, this paper assumes that $r_{ij}^{\left(l\right)}$ is a discrete random variable, which measures the extent to which the decision makers believe the own credit risk of firm *S*_*i*_ to be higher than that of *S*_*j*_ with a probability of ${p=p(r}_{ij}^{\left(l\right)})$.

#### 3.2.3 Construction of an FCJM of quantitative indicators

Based on their different influences on the own credit risk of firms in the supply chain, this paper classifies quantitative indicators into two types: positive and negative. For a certain quantitative indicator, the greater its value, the lower the corresponding credit risk, and the indicator is called a positive indicator. Otherwise, it is called a negative indicator. As the utility function can describe the assessor’s attitude toward and preference for risk, this section uses the utility function to measure the decision makers’ preferences for the assessment objects. When banks assess the risk of loan applicants, the decision makers are generally rational, prudent, and conservative, and have a risk-averse attitude to a certain extent. The lower the credit risk of an assessment object, the greater its utility, and the more the assessors prefer that assessment object.

Assume that *n* firms in the supply chain risk contagion network have *m* indicators (*X*_1_, *X*_2_,⋯,*X*_*m*_) for assessing their own credit risk. The utility function of the assessed is $\mu_{i}^{\left(l\right)}=\mu_{R}(X_{i}^{\left(l\right)})$, where $X_{i}^{\left(l\right)}$ is the value of the *l*th quantitative indicator of the *i*th firm, $\mu_{i}^{\left(l\right)}$ is the utility value corresponding to the *l*th quantitative indicator of the *i*th firm, and *i* = 1,2,⋯,*n*. By comparing the utility values $\mu_{i}^{\left(l\right)}$ and $\mu_{j}^{\left(l\right)}$ of different firms in the supply chain under the same quantitative indicator *X*_*l*_, we can determine the decision maker’s preference between assessment objects *S*_*i*_ and *S*_*j*_. Drawing on the study carried out by Tversky and Kahneman [25], we assume that the utility function of the assessment target is

$$\mu_{i}^{\left(l\right)}={\left(X_{i}^{\left(l\right)}\right)}^{\alpha },\;0<\alpha <1,$$ (6)

where *α* is the degree of concavity or convexity of the utility function based on prospect theory, reflecting decision makers’ different degrees of risk aversion towards gain and loss. The bigger *α* is, the more likely the decision maker is to take risks. The value of *α* can be obtained by assessing the risk-averse attitude of the decision makers.

To eliminate the influence of inconsistent quantitative indicators on the assessment results, the authors normalized the quantitative indicator *X*_*l*_ in this paper. If *X*_*l*_ is a positive quantitative indicator, then $\mu_{i}^{\left(l\right)}=\mu_{R}(X_{i}^{\left(l\right)})={\left(\frac{X_{i}^{\left(l\right)}-X_{Min}^{\left(l\right)}}{X_{Max}^{\left(l\right)}-X_{Min}^{\left(l\right)}}\right)}^{\alpha }$. If *X*_*l*_ is a negative quantitative indicator, then $\mu_{i}^{\left(l\right)}=\mu_{R}(X_{i}^{\left(l\right)})={\left(\frac{X_{Max}^{\left(l\right)}-X_{i}^{\left(l\right)}}{X_{Max}^{\left(l\right)}-X_{Min}^{\left(l\right)}}\right)}^{\alpha }$. Let $X_{Max}^{\left(l\right)}={Max\{X}_{1}^{\left(l\right)},X_{2}^{\left(l\right)},\cdots ,X_{n}^{\left(l\right)}\}$, $X_{Min}^{\left(l\right)}=Min{\{X}_{1}^{\left(l\right)},X_{2}^{\left(l\right)},\cdots ,X_{n}^{\left(l\right)}\}$, and *i* = 1,2,⋯,*n*. Therefore, $\mu_{ij}^{\left(l\right)}$ can be used to measure the decision maker’s preference for the own credit risk of firms *S*_*i*_ and *S*_*j*_ in the supply chain risk contagion network under the quantitative indicator *X*_*l*_:

$$\mu_{ij}^{\left(l\right)}=1-\frac{\mu_{i}^{\left(l\right)}}{\mu_{i}^{\left(l\right)}+\mu_{j}^{\left(l\right)}}=\frac{\mu_{j}^{\left(l\right)}}{\mu_{i}^{\left(l\right)}+\mu_{j}^{\left(l\right)}},i,j=1,2,\cdots ,n.$$ (7)

Eq (7) indicates that $0<\mu_{ij}^{\left(l\right)}<1$, $\mu_{ij}^{\left(l\right)}+\mu_{ji}^{\left(l\right)}=1$, $\mu_{ii}^{\left(l\right)}=0.5,$ and ∀*i*,*j* = 1,2,⋯,*n*. Thus, $\mu_{ij}^{\left(l\right)}$ can be understood as a fuzzy evaluation of the decision maker’s preference for the own credit risk of firms *S*_*i*_ and *S*_*j*_ under the quantitative indicator *X*_*l*_. When $\mu_{ij}^{\left(l\right)}=0.5$, it means the decision maker judges the own credit risk of *S*_*i*_ to be the same as that of *S*_*j*_; when $\mu_{ij}^{\left(l\right)}>0.5$, it means the decision maker judges the own credit risk of *S*_*i*_ to be higher than that of *S*_*j*_. The greater the $\mu_{ij}^{\left(l\right)}$, the more the decision maker considers the own credit risk of *S*_*i*_ to be higher than that of *S*_*j*_.

In summary, based on the quantitative indicator *X*_*l*_, the FCJM of firms in the supply chain risk contagion network can be expressed as a square matrix of order *n*:

$$U^{\left(l\right)}={\left(\mu_{ij}^{\left(l\right)}\right)}_{n\times n}.$$ (8)

### 3.3 FCJM consistency test and improvement

To ensure the credibility of the assessment results of the FCJM, this paper uses the eigenvector method to construct the consistency indicator $CR=CI/RI=\frac{\lambda_{Max}-n}{(n-1)RI}$ to check its consistency, where *RI* is the mean random consistency index [18]. For the FCJM of qualitative indicators, whose elements are discrete random variables $r_{ij}^{\left(l\right)}$, the consistency test method needs to be improved.

Given a qualitative indicator *X*_*l*_, if the probability distribution of the discrete random variable $r_{ij}^{\left(l\right)}$ is known, we can obtain the set of matrices ${H(R}^{\left(l\right)})$ consisting of all possible values of *R*^(*l*)^, and thereby obtain ${CR}_{R^{\left(l\right)}}$, which is all possible consistency indicators corresponding to the set of fuzzy comparison matrices ${H(R}^{\left(l\right)})$. The joint probability of the FCJM *R*^(*l*)^ is represented by $p(R^{\left(l\right)})=\prod_{r_{ij}^{\left(l\right)}{\in R}^{\left(l\right)}}p(r_{ij}^{\left(l\right)})$.

Check the consistency of the FCJM. If it fails to pass, then generate the improved FCJM. Drawing from Zhu et al. [26], this paper uses *E*(*CR*), the expectation of the consistency indicator *CR*, for consistency test:

$$E(CR)=\sum_{R^{\left(l\right)}\in {H(R}^{\left(l\right)})}{CR}_{R^{\left(l\right)}}p(R^{\left(l\right)}).$$ (9)

Eq (9) can evaluate the consistency of the FCJM of all qualitative and quantitative assessment indicators. When *E*(*CR*)<0.1, the FCJM is consistent; and when *E*(*CR*)>0.1, the FCJM is not consistent [26]. When the FCJM fails the consistency test, we can adjust its consistency so that the improved FCJM meets the conditions for consistency. Drawing on the study of Zhu et al. [26] and Lin et al. [27], we can employ a stochastic consistency improvement method to obtain a FCJM with acceptable consistency.

### 3.4 Weight calculation based on the eigenvector method

For firms’ own credit risk assessment indicator *X*_*l*_, its FCJM meeting the conditions for consistency is $R^{\left(l\right)}={\left(r_{ij}^{\left(l\right)}\right)}_{n\times n}$. As the probability distribution function of $r_{ij}^{\left(l\right)}$ is known, we can obtain the set of all possible matrices of the FCJM ${H(R}^{\left(l\right)}).\;{\forall R}^{\left(l\right)}\in {H(R}^{\left(l\right)}$). According to the eigenvector method, we can obtain the eigenvalues of all *R*^(*l*)^ in the matrix set ${H(R}^{\left(l\right)}$), and the eigenvectors corresponding to the eigenvalues $\omega_{R^{\left(l\right)}}=(\omega_{{1,R}^{\left(l\right)}},\omega_{2,R^{\left(l\right)}},\cdots ,\omega_{n,R^{\left(l\right)}})$. And then, we can obtain the maximum eigenvalue $\lambda_{Max}^{\left(l\right)}$ of ${H(R}^{\left(l\right)}$). Since the eigenvector $\omega_{R^{\left(l\right)}}$ measures the weight coefficients of *n* firms in the supply chain risk contagion network, we can rank the *n* firms according to the weight vector $\omega_{R^{\left(l\right)}}$.

Define the rank function of firm *S*_*i*_ regarding the assessment indicator *X*_*l*_ as

$${Rank}_{i}(\omega_{R^{\left(l\right)}})=1+\sum_{k=1}^{n}\theta (\omega_{{k,R}^{\left(l\right)}}>\omega_{{i,R}^{\left(l\right)}})=r,$$ (10)

When $\omega_{{k,R}^{\left(l\right)}}>\omega_{{i,R}^{\left(l\right)}}$, we assume $\theta (\omega_{{k,R}^{\left(l\right)}}>\omega_{{i,R}^{\left(l\right)}})=1$. When $\omega_{{k,R}^{\left(l\right)}}\leq \omega_{{i,R}^{\left(l\right)}}$, we assume $\theta (\omega_{{k,R}^{\left(l\right)}}\leq \omega_{{i,R}^{\left(l\right)}})=0$.

Define ${{Ζ}_{i}^{r}(R}^{\left(l\right)})\subseteq H(R^{\left(l\right)})$, which is the set of comparison judgement matrices of the firm *S*_*i*_ ordered by *r*, as

$${{Ζ}_{i}^{r}(R}^{\left(l\right)})=\{R^{\left(l\right)}:\;{Rank}_{i}(\omega_{R^{\left(l\right)}})=1+\sum_{k=1}^{n}\theta (\omega_{{k,R}^{\left(l\right)}}>\omega_{{i,R}^{\left(l\right)}})=r\}.$$ (11)

Thus, under firms’ own credit risk assessment indicator *X*_*l*_, the probability that the overall ranking of firm *S*_*i*_ is *r* for the set of fuzzy comparison judgment matrices ${H(R}^{\left(l\right)}$ is

$$p_{i,(l)}^{r}=\sum_{{{Ζ}_{i}^{r}(R}^{\left(l\right)})}p(R^{\left(l\right)})=\sum_{{{Ζ}_{i}^{r}(R}^{\left(l\right)})}\left[\prod_{r_{ij}^{\left(l\right)}{\in R}^{\left(l\right)}}p(r_{ij}^{\left(l\right)})\right],$$ (12)

where $p_{i,(l)}^{r}\in [0,\;1]$, and $\sum_{r=1}^{n}p_{i,(l)}^{r}=1$. The greater the $p_{i,(l)}^{r}$, the greater the probability that the ranking of firm *S*_*i*_ is *r*.

To obtain the overall probability of firm *S*_*i*_ for all possible ratings, we need to integrate the probabilities of each possible ranking of *S*_*i*_. Use the linear weight *b*^*r*^ = (*n*−*r*)/(*n*−1) to obtain the weighted sum of each ranking probability [28] and *n* is the order of the FCJM. Thus, the overall probability of firm *S*_*i*_ for all possible ratings is $\mu_{i}^{\left(l\right)}=\sum_{r=1}^{n}b^{r}p_{i,(l)}^{r}$). Let

$${p^{\left(l\right)}=(p}_{i}^{\left(l\right)}{)}_{n\times 1}={\left(p_{1}^{\left(l\right)},p_{2}^{\left(l\right)},\cdots ,p_{n}^{\left(l\right)}\right)}^{T},i=1,2,\cdots ,n,$$ (13)

where $p_{i}^{\left(l\right)}=\mu_{i}^{\left(l\right)}/(\sum_{i=1}^{n}\mu_{i}^{\left(l\right)})$, the vector *p*^(*l*)^ measures the weight of each firm in the supply chain risk contagion network with respect to firms’ own credit risk assessment indicator *X*_*l*_.

Thus, under (*X*_1_, *X*_2_,⋯,*X*_*m*_), which refers to the system of all the indicators for assessing firms’ own credit risk, the matrix of the weight vectors of all firms is *P* = (*p*^(1)^, *p*^(2)^,⋯,*p*^(*m*)^)^*T*^. According to the FCJM of the assessment indicator system *X*_1_, *X*_2_,⋯,*X*_*m*_ established by the decision maker, we can use the eigenvector method to obtain the weight vector of the assessment indicator system *X*_1_, *X*_2_,⋯,*X*_*m*_ as *ω* = (*ω*_1_, *ω*_2_,⋯,*ω*_*m*_).

By multiplying the weight vectors of firms and indicators, we can obtain a model for measuring the own credit risk of firms in the supply chain risk contagion network:

$$R_{s}=(\omega_{1},\omega_{2},\cdots ,\omega_{m}){\left(p^{\left(1\right)},p^{\left(2\right)},\cdots ,p^{\left(m\right)}\right)}^{T}=(R_{s1},\;R_{s2},\;\cdots ,\;R_{sn}).$$ (14)

## 4 Derivative model for assessing TCRC

In the supply chain network, whether there exists the contagion effect of credit risk among enterprises depends on whether there is a contagion path between risk sources. If there is no contagion path between supply chain enterprises, even if the credit risk of enterprises is very high, the credit risk between enterprises still does not have any contagion effect.

The risk score matrix *A* = (*a*_*ij*_)_*N*×*N*_, and

$$a_{ij}=\left\{ \begin{array}{c} {0<I}^{t}\leq 1,\;if\;there\;is\;an\;edge\;pointing\;from\;i\;to\;j\\ 0,\;otherwise \end{array}\right.,$$

where *I* is the line of trade credit extended by firm *S*_*j*_ to firm *S*_*i*_, and *t* is the corresponding duration of trade credit. *A* reveals the intensity of credit risk contagion among firms with trade credit relationships. To analyze the impact of trade credit on the contagion effect from a holistic perspective of the supply chain, we normalized the line of trade credit $I:\bar{I_{ij}}=\frac{I_{ij}}{\sum_{i=1}^{n}\sum_{j=1}^{n}B_{ij}}$, where *I*_*ij*_ is the line of trade credit extended by *S*_*j*_ to *S*_*i*_, and $\bar{I_{ij}}$ is the normalized line of trade credit.

If the maximum contagion distance *m* is a positive integer, then firms’ “(trade) credit risk contagion” *R*_*c*_ is calculated as $R_{c}=\sum_{i=1}^{m}A^{i}R_{s}$. If *m*→+∞, then $R_{c}=\underset{m\to +\infty }{lim}\sum_{i=1}^{m}A^{i}R_{s}$, where *R*_*c*_ measures the contagion effect of associated credit risk in the supply chain. Since firms’ credit risk is from two sources, namely their “own credit risk” *R*_*s*_ and “(trade) credit risk contagion” *R*_*c*_, we can assess the associated credit risk of firms in the supply chain risk contagion network as a whole according to Eq (3).

## 5 Case simulation and model evaluation

### 5.1 Credit risk assessment in automotive supply chain

**Fig 3:**
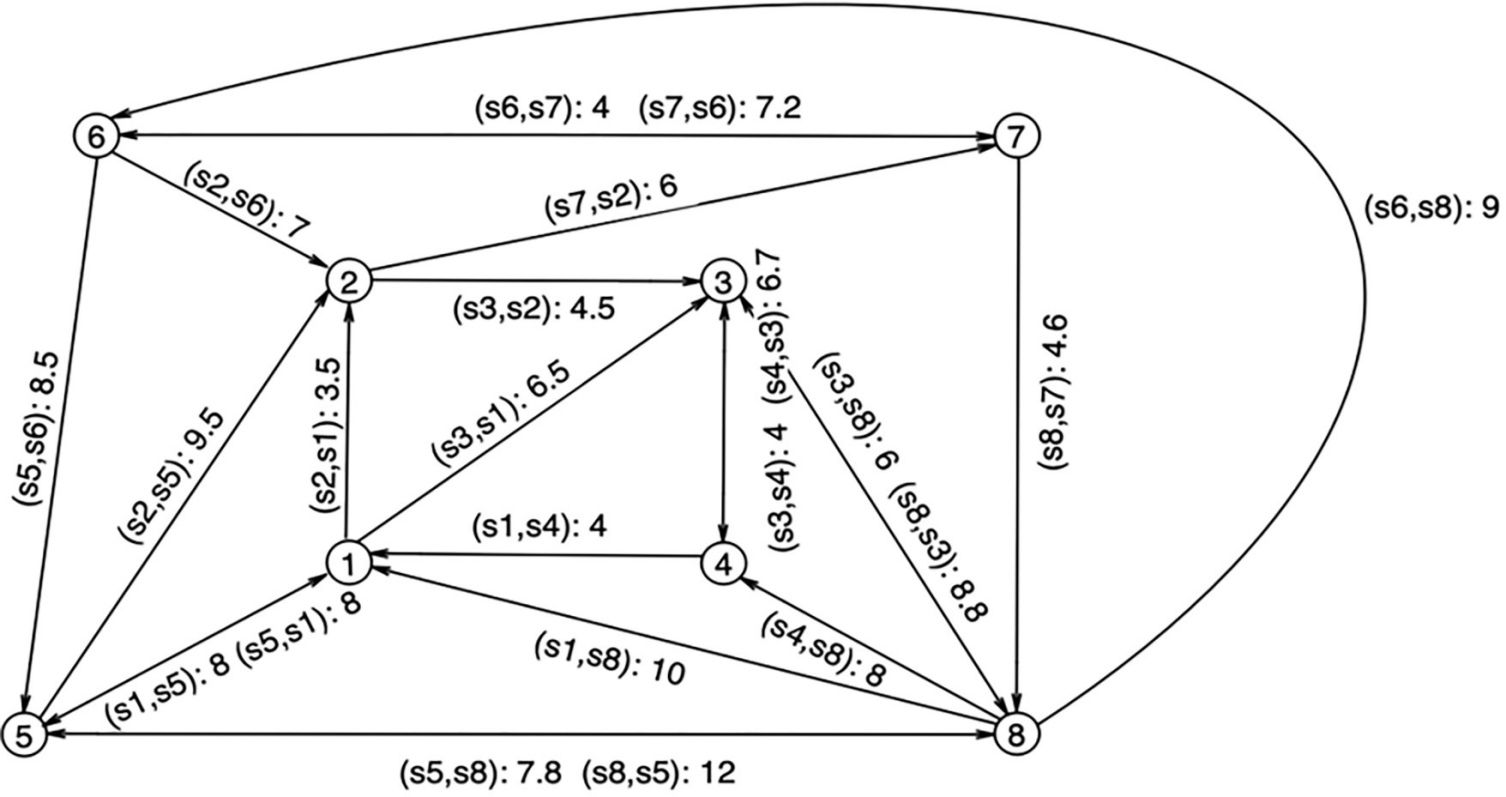
Risk contagion network of the automotive supply chain.

In recent years, there have been frequent “credit sales” in the automotive supply chain. In the meantime, trade credit has led to repeated group credit defaults, causing a credit crisis in the whole automotive supply chain. This paper studies the assessment of associated credit risk in the supply chain based on trade credit with the automotive supply chain as a case study. Specifically, the existing 8 firms *S*_1_, *S*_2_,⋯,*S*_8_ form the risk contagion network of the automotive supply chain. *S*_4_ is the core firm in the supply chain, and the remaining 7 firms are upstream and downstream firms. The trade credit relationships among these firms are shown in Fig 3. Assume that the duration of trade credit in the automotive supply chain risk contagion network are the same, and *t* = 1.

For each of the 31 credit risk assessment indicators in the automotive supply chain risk contagion network, we can obtain the corresponding FCJM and the weight vectors of firms under each assessment indicator. Take the qualitative indicator “industry characteristics of associated firms” (*X*_29_) for example, we construct a FCJM of the 8 firms in the automotive supply chain as follows:

$$R^{\left(29\right)}=[\begin{array}{cccccccc} 0.5 & 0.6 & 0.7 & 0.8 & 0.4 & 0.6 & 0.7 & 0.4\\ 0.4 & 0.5 & 0.6 & 0.8\;0.9 & 0.3\;0.4\;0.6 & 0.4 & 0.6 & 0.4\\ 0.3 & 0.4 & 0.5 & 0.6\;0.8 & 0.2 & 0.4 & 0.8 & 0.3\\ 0.2 & 0.1\;0.2 & 0.2\;0.4 & 0.5 & 0.1 & 0.3 & 0.6 & 0.2\\ 0.6 & 0.4\;0.6\;0.7 & 0.8 & 0.9 & 0.5 & 0.7 & 0.8 & 0.4\\ 0.4 & 0.6 & 0.6 & 0.7 & 0.3 & 0.5 & 0.6 & 0.4\\ 0.3 & 0.4 & 0.2 & 0.4 & 0.2 & 0.4 & 0.5 & 0.3\\ 0.6 & 0.6 & 0.7 & 0.8 & 0.6 & 0.6 & 0.7 & 0.5 \end{array}]$$

In *R*^(29)^, the probability distributions of random variables $r_{24}^{\left(29\right)},\;r_{25}^{\left(29\right)}\;and\;r_{34}^{\left(29\right)}$ are shown in Table 3 below:

**Table 3:** Probability distributions of $r_{24}^{\left(29\right)},\;r_{25}^{\left(29\right)},\;r_{34}^{\left(29\right)}$.

$r_{24}^{\left(29\right)}$	0.8	0.9	$r_{25}^{\left(29\right)}$	0.3	0.4	0.6	$r_{34}^{\left(29\right)}$	0.6	0.8
*p*	0.45	0.55	*p*	0.47	0.3	0.23	*p*	0.8	0.2

${H(R}^{\left(29\right)})$, which is the set of all possible matrices of the FCJM *R*^(29)^, contain 12 matrices, and the probability distribution of its consistency index *CR* is shown in Table 4 below:

**Table 4:** Probability distribution of consistency index *CR*.

*CR*	-0.4317	-0.4308	-0.4294	-0.4334	-0.4325	-0.4310
*p*	0.1692	0.108	0.0828	0.0423	0.027	0.0207
*CR*	-0.4329	-0.4320	-0.4307	-0.4347	-0.4339	-1.0784
*p*	0.2068	0.132	0.1012	0.0517	0.033	0.0253

It can be known from Table 4 that the expected consistency index of *R*^(29)^ is *E*(*CR*) = −0.4403<0.1, which means the FCJM *R*^(29)^ passes the consistency test. Therefore, the assessment results of the weights it has constructed are relevant.

According to Es (10)-(13), we can obtain the linear and reciprocal weights of *S*_1_, *S*_2_,⋯,*S*_8_ as follows (see Table 5).

**Table 5:** Weights of *S*_1_, *S*_2_,⋯,*S*_8_ under the linear weighting coefficient.

	Linear weight *b*^*r*^ = (*n*−*r*)/(*n*−1)	
Firm	$\mu_{i}^{\left(29\right)}$	$p_{i}^{\left(29\right)}$
*S*_1_	0.7143	0.1786
*S*_2_	0.4661	0.1165
*S*_3_	0.2857	0.0714
*S*_4_	0.012	0.045
*S*_5_	0.9671	0.2418
*S*_6_	0.5339	0.1335
*S*_7_	0.1429	0.0357
*S*_8_	0.89	0.2225

Thus, the weight vectors of *S*_1_, *S*_2_,⋯,*S*_8_ under linear weight are:

$$p^{\left(29\right)}={\left(0.1786,\;0.1165,\;0.0714,\;0,\;0.2418,0.1335,0.0357,\;0.2225\right)}^{T}.$$

The order of the firms ranked according to *X*_29_ is:

$$S_{4}≻S_{7}≻S_{3}≻S_{2}≻S_{6}≻S_{1}≻S_{8}≻S_{5}.$$

When calculating the utility function of quantitative indicators, we assume that *α* = 1/2. Thus, we can obtain the weight vectors of *S*_1_, *S*_2_,⋯,*S*_8_ under all 31 assessment indicators as *p*^(1)^、*p*^(2)^、*p*^(3)^,⋯, *p*^(31)^. See Table 1 in S1 Appendix for specific data. To obtain the weight vectors of *X*_1_, *X*_2_, ⋯, *X*_31_, which belong to the system of indicators for assessing firms’ own credit risk in the automotive supply chain, we now construct the preference relation matrix *R*_*X*_ of the assessment indicators (see S1 Appendix). If its maximum eigenvalue *λ*_*Max*_ = 15.1085, and consistency index *CR* = −0.3332<0.1, then *R*_*X*_ passes the consistency test. We then use the eigenvector method to obtain the weight vectors of the assessment indicator system:

*ω* = (0.0287, 0.0283, 0.0294, 0.0304, 0.0327, 0.03, 0.0485, 0.029, 0.0264, 0.0323, 0.031, 0.0314, 0.0281, 0.027, 0.0297, 0.0382, 0.0407, 0.0372, 0.0352, 0.0337, 0.037, 0.0367, 0.037, 0.0377, 0.0378, 0.0272, 0.0261, 0.0272, 0.0327, 0.0258, 0.0263).

Using Eq (3) that measures the own credit risk of firms in the supply chain risk contagion network, we can obtain firms’ own credit risk *R*_*s*_ in the automotive supply chain risk contagion network as

$$R_{s}=(0.2094,0.1089,0.106,0.0156,0.1654,0.12,0.109,0.1589),$$ (15)

According to Eq (15), the order of the “own credit risk” of the firms in the automotive supply chain is as follows:

$$S_{4}≻S_{3}≻S_{2}≻S_{7}≻S_{6}≻S_{8}≻S_{5}≻S_{1}.$$

It can be seen that the own credit risk of the core firm *S*_4_ is 0.01564, which is the lowest in the automotive supply chain; while the own credit risk of firm *S*_1_ is 0.20937, which is the highest in the automotive supply chain.

The risk score matrix *A* in the automotive supply chain risk contagion network is measured based on the path and degree of trade credit influence. We then use Eq (3) to obtain the risk score matrix for the automotive supply chain risk contagion network:

$$A=[\begin{array}{cccccccc} 0 & 0 & 0 & 0.0355 & 0.0709 & 0 & 0 & 0.0887\\ 0.031 & 0 & 0 & 0 & 0.0842 & 0.0621 & 0 & 0\\ 0.0576 & 0.0399 & 0 & 0.0355 & 0 & 0 & 0 & 0.0532\\ 0 & 0 & 0.0594 & 0 & 0 & 0 & 0 & 0.2\\ 0.6 & 0.4 & 0.8 & 0.9 & 0.5 & 0.7 & 0.8 & 0.0709\\ 0.071 & 0 & 0 & 0 & 0 & 0.0754 & 0 & 0.0691\\ 0 & 0.0532 & 0 & 0 & 0 & 0.0638 & 0 & 0\\ 0 & 0 & 0.0781 & 0 & 0.1064 & 0 & 0.0408 & 0 \end{array}]$$

Since *A*^8^ = 0, the “(trade) credit risk contagion” *R*_*c*_ of the firms in the automotive supply chain is:

$$R_{c}=\sum_{i=1}^{7}A^{i}\;R_{s}=(0.0301,0.0126,0.0228,0.014,0.057,0.0359,0.017,0.0648)$$ (16)

Eq (16) indicates that all the firms in the automotive supply chain are subject to credit risk contagion from other associated firms in the automotive supply chain risk contagion network, resulting in different degrees of credit risk increase for each firm. Besides, due to its strong risk resistance capacity, the core firm *S*_4_ suffers the least from the credit risk of other associated firms in the automotive supply chain risk contagion network, with the “(trade) credit risk contagion” being only 0.014. As firm *S*_8_ has extended more trade credit to other firms and the amount is relatively large, together with its own weak risk resistance capacity, it is subject to relatively strong credit risk contagion from other associated firms and has the largest “(trade) credit risk contagion” of 0.0648.

It is easily observed that the credit risk of all the firms in the automotive supply chain is

TR=(0.2395,0.1215,0.1287,0.0296,0.2224,0.156,0.1259,0.2237). (17)

Eq (17) suggests that the credit risk ranking of the eight firms in the automotive supply chain is as follows:

$$S_{4}≻S_{2}≻S_{7}≻S_{3}≻S_{6}≻S_{5}≻S_{8}≻S_{1}.$$

It can be seen that the core firm *S*_4_ in the automotive supply chain has the lowest credit risk, with a probability of default of only 0.0296, indicating that its credit status is good, and that the risk of default faced by banks in extending credit to it is low. The credit risk of firm *S*_1_ in the automotive supply chain is the highest, with a probability of default of 0.2395, indicating that its credit quality is relatively poor, and that the risk of default faced by banks in extending credit to it is relatively high. The assessment results of the credit risk of the firms in the automotive supply chain risk contagion network show that the overall credit risk of the firms in the automotive supply chain risk contagion network is low, indicating that both the firms in the automotive supply chain and the supply chain itself are in sound operation, and that the possibility of a systemic risk outbreak in the supply chain is low.

### 5.2 Credit risk model evaluation in petrochemical supply chain

With the gradual increase of oil prices in the international market recently, the capital demand of petrochemical industry enterprises is increasing day by day, which is a field with huge demand for supply chain finance [29]. To help the development of the petrochemical industry, Industrial Bank issued a total of 1.989 billion yuan of loans to 115 upstream enterprises of HENG LI Petrochemical. With the extensive development of the supply chain finance business and the frequent occurrence of the default behavior of various enterprises, the financial institutions must develop risk assessment and control technology for petrochemical enterprises in the supply chain finance industry. For example, Shandong Dahai Group and Shandong Jinmao Textile & Chemical Group are both local oil refiners in Shandong Province. In 2019, the two companies with asset relationship entered the bankruptcy reorganization process, which highlighted the problem of credit risk contagion in the refining industry.

A Petrochemical Company *P*_1_ is a local oil refining enterprise, and its upstream supplier *P*_2_ is a subsidiary of China Petroleum and Chemical Company Limited. The supplier *P*_2_ provides the Petrochemical Company *P*_1_ with stable crude oil supply according to crude oil indicators every month, and *P*_1_ refines and produces. The Petrochemical Company *P*_1_ trades with its upstream supplier *P*_2_ in the form of trade credit. Because the upstream supplier *P*_2_ has the fund demand for purchasing raw materials, it applies for supply chain finance based on the receivable payments from the bank and provides *P*_1_ with crude oil sales based on trade credit.

Xiong et al. [20] made an initial score on the credit status of the Petrochemical Company *P*_1_ and its upstream supplier *P*_2_ according to their corporate status, financial status, and transaction status. Taking a single enterprise as the evaluation object, they built a credit risk evaluation index system of 21 indicators and 4 dimensions including applicant qualification, counterparty qualification, assets under financing, and supply chain operation. They used principal component analysis to select representative independent variables to reduce the correlation between candidate indicators. Finally, the default probability is calculated by the logistic regression model.

According to the algorithm proposed by Xiong et al. [20], the 5 principal component factors of the 21 indicators are obtained by the formulas *F*_1_ = −0.91364, *F*_2_ = 1.75525, *F*_3_ = −0.71896, *F*_8_ = −0.00172, *F*_9_ = −0.76100. Substituting the results of the five principal component factors into the logistic regression equation, it can be concluded that the probability *P* value of *P*_2_ is:

$$p_{2}=\frac{1}{1+e^{-(1.286+0.976F_{1}+1.676F_{2}+1.154F_{3}+1.476F_{8}+0.783F_{9})}}=0.87079.$$

We can see from their model that the probability *P* value of the upstream supplier *P*_2_ is 87.079%. The evaluation results show that the probability of keeping the promises of the upstream supplier *P*_2_ is 87.079% and the default probability of *P*_2_ is 12.921%. According to their credit risk assessment model, the credit status of the upstream supplier *P*_2_ is good, and the bank should provide loan to *P*_2_.

However, by using the credit risk assessment model we constructed, the credit risk of the upstream supplier *P*_2_ rises significantly to 0.74002. By using our model, we consider the contagion effect of the Petrochemical Company *P*_1_’s credit risk on the upstream supplier *P*_2_’s credit risk. Through the similar evaluation method, we can obtain the probability *P* value of *P*_1_ is:

$$p_{1}=\frac{1}{1+e^{-(0.694+0.690F_{1}^{\prime }+0.499F_{2}^{\prime })}}=0.38919.$$

Therefore, the own credit risk of the Petrochemical Company *P*_1_ is 1−0.38919 = 0.61081, and the value matrix of trade credit risk between the Petrochemical Company *P*_1_ and the upstream supplier *P*_2_ is *A* = 1, and the risk distance *m* = 1, then the “(trade) credit risk contagion” of the Petrochemical Company *P*_1_ to the upstream supplier *P*_2_ is *R*_*C*_ = (1−0.38919)*A^*m*^ = 0.61081. By using the credit risk assessment model we constructed, the credit risk of the *P*_1_ rises to *TR*_*P*1_ = 0.12921+0.61081 = 0.74002. The results indicate that after considering the contagion effect of credit risk in the petrochemical supply chain, the probability of keeping promise of *P*_2_ is only 25.998%, and the bank is difficult to grant credit to *P*_2_ due to its poor credit status. Thus, the credit risk assessment approach proposed in this paper is more scientific and accurate due to the incorporation of the risk factors of the enterprise itself and the risk contagion factors of the counterparty.

In practice, it is common for enterprises in supply chain to default due to the contagion effect of credit risk. For example, due to the bad debts of the upstream and downstream auto parts supplier chain of about 50-billion-yuan, Cheetah Automobile, Zotye Automobile, Hawtai Automobile, and Li Fan Automobile entered the bankruptcy procedure at the end of 2019. In another example, Gionee Mobile Phone defaulted on about 500 million yuan in debt owed to its small and medium-sized suppliers due to a break in its capital chain. Finally, the bankruptcy of Gionee mobile phone led to more than 20 small and medium-sized suppliers also faced the risk of bankruptcy [30]. Therefore, in the supply chain scenario, financial institutions should consider not only the credit risk of the enterprise itself, but also the contagion effect of credit risk based on trade credit in the supply chain [31].

## 6 Conclusion

The traditional credit risk assessment method used by banks fails to consider the contagion of credit risk among associated firms, which leads to banks’ unscientific assessment results of the credit risk of firms applying for loans as well as unreasonable credit decisions and risk control measures, thus increasing the credit risk faced by banks themselves. This paper classifies the credit risk of firms in the supply chain into two types, namely firms’ “own credit risk” and “(trade) credit risk contagion”. It constructs a basic model for assessing firms’ “own credit risk” and a derivative model for assessing “(trade) credit risk contagion” in the context of the risk contagion network formed by trade credit. Then it integrates the two types of assessment models and carries out a comprehensive assessment of the credit risk of firms in the supply chain risk contagion network.

This paper also offers important managerial insights. From the perspective of the supply chain, this paper innovatively constructs a theoretical model of credit risk assessment, and it measures the credit risk of enterprises in the supply chain network as the coupling effect of enterprises’ “own credit risk” and “(trade) credit risk contagion,” which makes up for the deficiency of banks’ focusing on their own factors in enterprise credit risk assessment. At the same time, it improves the accuracy of credit risk assessment results. First, based on the evaluation model, this paper introduces an index system to evaluate the enterprise’s own credit risk under the supply chain environment. Second, a fuzzy credit risk assessment model is constructed based on fuzzy preference theory, which is used to evaluate the enterprise’s “own credit risk.” On this basis, the enterprise “(trade) credit risk contagion” is evaluated. Finally, based on the evaluation results of “own credit risk” and “(trade) credit risk contagion” of enterprises, the credit risk of enterprises in the supply chain risk contagion network is measured. In addition, to test the adaptability and effectiveness of the model constructed in this paper, the credit risk assessment method of supply chain association based on TCRC is used to evaluate the credit risk of automobile supply chain enterprises and petrochemical supply chain enterprises, respectively, verifying the practicability and operability of the model.

The research results show that the TCRC-based method for assessing the associated credit risk in the supply chain is closer to the actual operating environment of the supply chain as it factors in the credit risk contagion among the firms in the chain, and its assessment results are more scientific. The assessment method put forward in this paper has not only comprehensively and effectively assessed the credit risk of firms in the supply chain risk contagion network and revealed the contagion effect of associated credit risk in the supply chain. They can also help banks reduce or even eliminate the negative impact of associated credit risk in the supply chain and thereby reduce their potential losses.

This paper still has some limitations on the evaluation method of associated credit risk in the supply chain risk contagion network. This paper constructs the corresponding evaluation model only for the risk contagion network constituted by trade credit. The transmission channels of credit risk between enterprises in the supply chain may be complex, so it is a great challenge to study the evaluation model of credit risk in different correlative situations. This research is only a preliminary exploration and attempt. In the future, according to the economic correlation and social correlation between the supply chain enterprises, we will build corresponding credit risk assessment models to evaluate the credit risk of enterprises under different supply chain correlation situations.

## Supporting information

S1 Appendix (DOCX)
