# Supplementary material for: Assessment of associated credit risk in the supply chain based on trade credit risk contagion
Source: PLoS One. 2023 Feb 16;18(2):e0281616. doi: 10.1371/journal.pone.0281616 (PMC9934404; doi:10.1371/journal.pone.0281616)
Supplement: S1 Appendix — (DOCX) [file pone.0281616.s001.docx]

**Appendices:**

**Table 1: Weight vectors of firms in the supply chain risk contagion network under the indicators for assessing firms’ own credit risk**

| Weight vector | $S_{1}$ | $S_{2}$ | $S_{3}$ | $S_{4}$ | $S_{5}$ | $S_{6}$ | $S_{7}$ | $S_{8}$ |
| --- | --- | --- | --- | --- | --- | --- | --- | --- |
| $X_{1}$ | 0.1615 | 0.1159 | 0.064 | 0.0454 | 0.2524 | 0.129 | 0.0984 | 0.1334 |
| $X_{2}$ | 0.1251 | 0.128 | 0.128 | 0.1155 | 0.128 | 0.1251 | 0.1251 | 0.1251 |
| $X_{3}$ | 0.1112 | 0.084 | 0.1112 | 0.1112 | 0.276 | 0.084 | 0.1112 | 0.1112 |
| $X_{4}$ | 0.1111 | 0.1112 | 0.084 | 0.084 | 0.3035 | 0.1111 | 0.084 | 0.1111 |
| $X_{5}$ | 0.1572 | 0.1324 | 0.1426 | 0 | 0.1674 | 0.1426 | 0.0952 | 0.1626 |
| $X_{6}$ | 0.1666 | 0.142 | 0.1276 | 0 | 0.172 | 0.1276 | 0.104 | 0.1602 |
| $X_{7}$ | 0.18 | 0.1235 | 0.18 | 0 | 0.2131 | 0.1235 | 0 | 0.18 |
| $X_{8}$ | 0.1532 | 0.1401 | 0.1401 | 0 | 0.1734 | 0.1243 | 0.1048 | 0.1641 |
| $X_{9}$ | 0.1739 | 0.1724 | 0 | 0.0178 | 0.2262 | 0.1421 | 0.0938 | 0.1739 |
| $X_{10}$ | 0.171 | 0.0439 | 0.1061 | 0 | 0.2741 | 0.1548 | 0.0661 | 0.1841 |
| $X_{11}$ | 0.1339 | 0.1068 | 0.1301 | 0.0231 | 0.154 | 0.1572 | 0.1475 | 0.1475 |
| $X_{12}$ | 0.1667 | 0.1667 | 0.1667 | 0 | 0.1667 | 0 | 0.1667 | 0.1667 |
| $X_{13}$ | 0.173 | 0.144 | 0.0966 | 0 | 0.173 | 0.144 | 0.0966 | 0.173 |
| $X_{14}$ | 0.0893 | 0.1504 | 0.0958 | 0 | 0.2493 | 0.1355 | 0.1185 | 0.1611 |
| $X_{15}$ | 0.2131 | 0.158 | 0 | 0.0414 | 0.2062 | 0.1669 | 0.0185 | 0.196 |
| $X_{16}$ | 0.1561 | 0.1561 | 0.1234 | 0 | 0.1667 | 0.1411 | 0.1006 | 0.1561 |
| $X_{17}$ | 0.2392 | 0 | 0.2392 | 0 | 0.2823 | 0 | 0 | 0.2392 |
| $X_{18}$ | 0.1685 | 0.1033 | 0.1025 | 0 | 0.1795 | 0.1408 | 0.1509 | 0.1545 |
| $X_{19}$ | 0.1743 | 0.1291 | 0.1453 | 0 | 0.2543 | 0.0499 | 0.1529 | 0.0943 |
| $X_{20}$ | 0.1652 | 0.1045 | 0.1131 | 0 | 0.1734 | 0.142 | 0.1474 | 0.1544 |
| $X_{21}$ | 0.0983 | 0.1016 | 0.1101 | 0 | 0.2676 | 0.1383 | 0.1664 | 0.1176 |
| $X_{22}$ | 0.1589 | 0.1015 | 0.1095 | 0 | 0.1674 | 0.1457 | 0.169 | 0.148 |
| $X_{23}$ | 0.1132 | 0.0984 | 0.0559 | 0 | 0.3632 | 0.0917 | 0.1143 | 0.1632 |
| $X_{24}$ | 0.1749 | 0.1078 | 0 | 0.0199 | 0.1718 | 0.1468 | 0.1948 | 0.1839 |
| $X_{25}$ | 0.1775 | 0.10367 | 0 | 0.0154 | 0.2757 | 0.1373 | 0.2039 | 0.0866 |
| $X_{26}$ | 0.1481 | 0.109 | 0.1105 | 0 | 0.1674 | 0.141 | 0.1759 | 0.1481 |
| $X_{27}$ | 0.1628 | 0.1051 | 0.1179 | 0 | 0.1628 | 0.135 | 0.1536 | 0.1628 |
| $X_{28}$ | 0.1811 | 0.1068 | 0.1299 | 0 | 0.1889 | 0.1259 | 0.0863 | 0.1811 |
| $X_{29}$ | 0.1225 | 0.0783 | 0.0625 | 0.0459 | 0.3252 | 0.087 | 0.0525 | 0.226 |
| $X_{30}$ | 0.1708 | 0.1056 | 0.106 | 0 | 0.1708 | 0.136 | 0.1552 | 0.1556 |
| $X_{31}$ | 0.197 | 0 | 0.1367 | 0 | 0.1664 | 0.1367 | 0.1664 | 0.197 |

**Preference relations matrix (**$\boldsymbol{R}_{\boldsymbol{X}}$**) of the indicators for assessing the own credit risk of firms in the automotive supply chain**

|  | X1 | X2 | X3 | X4 | X5 | X6 | X7 | X8 | X9 | X10 | X11 | X12 | X13 | X14 | X15 | X16 | X17 | X18 | X19 | X20 | X21 | X22 | X23 | X24 | X25 | X26 | X27 | X28 | X29 | X30 | X31 |
| --- | --- | --- | --- | --- | --- | --- | --- | --- | --- | --- | --- | --- | --- | --- | --- | --- | --- | --- | --- | --- | --- | --- | --- | --- | --- | --- | --- | --- | --- | --- | --- |
| X1 | 0.5 | 0.6 | 0.6 | 0.5 | 0.4 | 0.4 | 0.5 | 0.6 | 0.7 | 0.3 | 0.5 | 0.4 | 0.2 | 0.4 | 0.4 | 0.3 | 0.3 | 0.4 | 0.4 | 0.4 | 0.3 | 0.4 | 0.4 | 0.5 | 0.5 | 0.4 | 0.3 | 0.6 | 0.3 | 0.5 | 0.6 |
| X2 | 0.4 | 0.5 | 0.4 | 0.4 | 0.5 | 0.6 | 0.5 | 0.5 | 0.5 | 0.4 | 0.4 | 0.4 | 0.5 | 0.5 | 0.3 | 0.3 | 0.4 | 0.4 | 0.3 | 0.3 | 0.4 | 0.4 | 0.4 | 0.4 | 0.3 | 0.5 | 0.5 | 0.5 | 0.5 | 0.5 | 0.5 |
| X3 | 0.4 | 0.6 | 0.5 | 0.5 | 0.5 | 0.5 | 0.6 | 0.4 | 0.6 | 0.3 | 0.6 | 0.5 | 0.5 | 0.5 | 0.4 | 0.4 | 0.4 | 0.4 | 0.3 | 0.4 | 0.3 | 0.4 | 0.4 | 0.4 | 0.3 | 0.5 | 0.5 | 0.5 | 0.4 | 0.5 | 0.4 |
| X4 | 0.5 | 0.6 | 0.5 | 0.5 | 0.5 | 0.5 | 0.6 | 0.5 | 0.6 | 0.4 | 0.6 | 0.5 | 0.5 | 0.7 | 0.4 | 0.4 | 0.4 | 0.4 | 0.3 | 0.4 | 0.3 | 0.4 | 0.4 | 0.4 | 0.3 | 0.5 | 0.5 | 0.5 | 0.4 | 0.5 | 0.4 |
| X5 | 0.6 | 0.5 | 0.5 | 0.5 | 0.5 | 0.5 | 0.5 | 0.5 | 0.5 | 0.6 | 0.4 | 0.4 | 0.7 | 0.6 | 0.4 | 0.5 | 0.5 | 0.4 | 0.4 | 0.4 | 0.3 | 0.3 | 0.4 | 0.5 | 0.5 | 0.6 | 0.6 | 0.5 | 0.7 | 0.6 | 0.6 |
| X6 | 0.6 | 0.4 | 0.5 | 0.5 | 0.5 | 0.5 | 0.6 | 0.5 | 0.5 | 0.6 | 0.4 | 0.4 | 0.6 | 0.6 | 0.4 | 0.4 | 0.3 | 0.4 | 0.4 | 0.3 | 0.3 | 0.3 | 0.4 | 0.4 | 0.5 | 0.5 | 0.5 | 0.5 | 0.5 | 0.4 | 0.5 |
| X7 | 0.5 | 0.5 | 0.4 | 0.4 | 0.5 | 0.4 | 0.5 | 0.6 | 0.6 | 0.8 | 0.9 | 0.9 | 0.6 | 0.7 | 0.7 | 0.9 | 1 | 1 | 0.9 | 0.9 | 0.9 | 0.8 | 0.8 | 0.8 | 0.8 | 0.9 | 0.7 | 0.7 | 1 | 0.7 | 0.6 |
| X8 | 0.4 | 0.5 | 0.6 | 0.5 | 0.5 | 0.5 | 0.4 | 0.5 | 0.7 | 0.4 | 0.6 | 0.6 | 0.6 | 0.5 | 0.4 | 0.4 | 0.4 | 0.4 | 0.3 | 0.3 | 0.4 | 0.4 | 0.2 | 0.3 | 0.3 | 0.4 | 0.5 | 0.5 | 0.4 | 0.5 | 0.5 |
| X9 | 0.3 | 0.6 | 0.4 | 0.4 | 0.5 | 0.5 | 0.4 | 0.3 | 0.5 | 0.3 | 0.4 | 0.4 | 0.5 | 0.5 | 0.3 | 0.3 | 0.3 | 0.3 | 0.4 | 0.4 | 0.3 | 0.4 | 0.4 | 0.4 | 0.3 | 0.5 | 0.5 | 0.5 | 0.4 | 0.5 | 0.4 |
| X10 | 0.7 | 0.6 | 0.7 | 0.6 | 0.4 | 0.4 | 0.2 | 0.6 | 0.7 | 0.5 | 0.6 | 0.6 | 0.8 | 0.6 | 0.6 | 0.5 | 0.5 | 0.4 | 0.4 | 0.4 | 0.4 | 0.3 | 0.4 | 0.4 | 0.3 | 0.5 | 0.5 | 0.5 | 0.5 | 0.5 | 0.5 |
| X11 | 0.5 | 0.6 | 0.4 | 0.4 | 0.6 | 0.6 | 0.1 | 0.4 | 0.6 | 0.4 | 0.5 | 0.5 | 0.5 | 0.6 | 0.5 | 0.5 | 0.4 | 0.4 | 0.5 | 0.5 | 0.5 | 0.4 | 0.4 | 0.4 | 0.4 | 0.7 | 0.7 | 0.5 | 0.4 | 0.5 | 0.5 |
| X12 | 0.6 | 0.5 | 0.5 | 0.5 | 0.6 | 0.6 | 0.1 | 0.4 | 0.6 | 0.4 | 0.5 | 0.5 | 0.5 | 0.6 | 0.5 | 0.5 | 0.4 | 0.4 | 0.5 | 0.5 | 0.5 | 0.4 | 0.4 | 0.4 | 0.4 | 0.7 | 0.7 | 0.5 | 0.4 | 0.5 | 0.5 |
| X13 | 0.8 | 0.5 | 0.5 | 0.5 | 0.3 | 0.4 | 0.4 | 0.4 | 0.5 | 0.2 | 0.5 | 0.5 | 0.5 | 0.5 | 0.5 | 0.4 | 0.3 | 0.4 | 0.4 | 0.4 | 0.4 | 0.4 | 0.3 | 0.4 | 0.3 | 0.3 | 0.5 | 0.5 | 0.4 | 0.5 | 0.5 |
| X14 | 0.6 | 0.7 | 0.5 | 0.3 | 0.4 | 0.4 | 0.3 | 0.5 | 0.5 | 0.4 | 0.4 | 0.4 | 0.5 | 0.5 | 0.3 | 0.3 | 0.3 | 0.4 | 0.4 | 0.3 | 0.3 | 0.4 | 0.4 | 0.4 | 0.4 | 0.5 | 0.5 | 0.5 | 0.5 | 0.5 | 0.5 |
| X15 | 0.6 | 0.7 | 0.6 | 0.6 | 0.6 | 0.6 | 0.3 | 0.6 | 0.7 | 0.4 | 0.5 | 0.5 | 0.5 | 0.7 | 0.5 | 0.4 | 0.3 | 0.3 | 0.3 | 0.3 | 0.4 | 0.3 | 0.2 | 0.4 | 0.4 | 0.5 | 0.5 | 0.4 | 0.3 | 0.5 | 0.5 |
| X16 | 0.7 | 0.6 | 0.6 | 0.6 | 0.5 | 0.6 | 0.1 | 0.6 | 0.7 | 0.5 | 0.5 | 0.5 | 0.6 | 0.7 | 0.6 | 0.5 | 0.5 | 0.6 | 0.6 | 0.5 | 0.6 | 0.5 | 0.5 | 0.5 | 0.6 | 0.8 | 0.9 | 0.8 | 0.5 | 0.8 | 0.9 |
| X17 | 0.7 | 0.6 | 0.6 | 0.6 | 0.5 | 0.7 | 0 | 0.6 | 0.7 | 0.5 | 0.6 | 0.6 | 0.7 | 0.7 | 0.7 | 0.5 | 0.5 | 0.8 | 0.6 | 0.6 | 0.7 | 0.7 | 0.5 | 0.5 | 0.5 | 0.8 | 0.9 | 0.9 | 0.8 | 0.9 | 0.7 |
| X18 | 0.6 | 0.7 | 0.6 | 0.6 | 0.6 | 0.6 | 0 | 0.6 | 0.7 | 0.6 | 0.6 | 0.6 | 0.6 | 0.6 | 0.7 | 0.4 | 0.2 | 0.5 | 0.5 | 0.5 | 0.6 | 0.4 | 0.5 | 0.5 | 0.5 | 0.8 | 0.8 | 0.7 | 0.8 | 0.9 | 0.9 |
| X19 | 0.6 | 0.7 | 0.7 | 0.7 | 0.6 | 0.6 | 0.1 | 0.7 | 0.6 | 0.6 | 0.5 | 0.5 | 0.6 | 0.6 | 0.7 | 0.4 | 0.4 | 0.5 | 0.5 | 0.5 | 0.4 | 0.4 | 0.5 | 0.5 | 0.5 | 0.6 | 0.6 | 0.6 | 0.5 | 0.6 | 0.7 |
| X20 | 0.6 | 0.6 | 0.6 | 0.6 | 0.6 | 0.7 | 0.1 | 0.7 | 0.6 | 0.6 | 0.5 | 0.5 | 0.6 | 0.7 | 0.7 | 0.5 | 0.4 | 0.5 | 0.5 | 0.5 | 0.4 | 0.5 | 0.4 | 0.4 | 0.4 | 0.5 | 0.5 | 0.5 | 0.5 | 0.5 | 0.5 |
| X21 | 0.7 | 0.6 | 0.7 | 0.7 | 0.7 | 0.7 | 0.1 | 0.6 | 0.7 | 0.6 | 0.5 | 0.5 | 0.6 | 0.7 | 0.6 | 0.4 | 0.3 | 0.4 | 0.6 | 0.6 | 0.5 | 0.5 | 0.5 | 0.5 | 0.5 | 0.7 | 0.8 | 0.8 | 0.5 | 0.7 | 0.7 |
| X22 | 0.6 | 0.6 | 0.6 | 0.6 | 0.7 | 0.7 | 0.2 | 0.6 | 0.6 | 0.7 | 0.6 | 0.6 | 0.6 | 0.6 | 0.7 | 0.5 | 0.3 | 0.6 | 0.6 | 0.5 | 0.5 | 0.5 | 0.4 | 0.3 | 0.4 | 0.6 | 0.7 | 0.7 | 0.7 | 0.7 | 0.7 |
| X23 | 0.6 | 0.6 | 0.6 | 0.6 | 0.6 | 0.6 | 0.2 | 0.8 | 0.6 | 0.6 | 0.6 | 0.6 | 0.7 | 0.6 | 0.8 | 0.5 | 0.5 | 0.5 | 0.5 | 0.6 | 0.5 | 0.6 | 0.5 | 0.5 | 0.5 | 0.4 | 0.4 | 0.5 | 0.5 | 0.8 | 0.8 |
| X24 | 0.5 | 0.7 | 0.6 | 0.6 | 0.5 | 0.6 | 0.2 | 0.7 | 0.6 | 0.6 | 0.6 | 0.6 | 0.6 | 0.6 | 0.6 | 0.5 | 0.5 | 0.5 | 0.5 | 0.6 | 0.5 | 0.7 | 0.5 | 0.5 | 0.5 | 0.7 | 0.7 | 0.8 | 0.5 | 0.7 | 0.8 |
| X25 | 0.5 | 0.5 | 0.7 | 0.7 | 0.5 | 0.5 | 0.2 | 0.7 | 0.7 | 0.7 | 0.6 | 0.6 | 0.7 | 0.6 | 0.6 | 0.4 | 0.5 | 0.5 | 0.5 | 0.6 | 0.5 | 0.6 | 0.5 | 0.5 | 0.5 | 0.8 | 0.8 | 0.6 | 0.6 | 0.8 | 0.7 |
| X26 | 0.6 | 0.5 | 0.5 | 0.5 | 0.4 | 0.5 | 0.1 | 0.6 | 0.5 | 0.5 | 0.3 | 0.3 | 0.7 | 0.5 | 0.5 | 0.2 | 0.2 | 0.2 | 0.4 | 0.5 | 0.3 | 0.4 | 0.6 | 0.3 | 0.2 | 0.5 | 0.5 | 0.5 | 0.5 | 0.5 | 0.5 |
| X27 | 0.7 | 0.5 | 0.5 | 0.5 | 0.4 | 0.5 | 0.3 | 0.5 | 0.5 | 0.5 | 0.3 | 0.3 | 0.5 | 0.5 | 0.5 | 0.1 | 0.1 | 0.2 | 0.4 | 0.5 | 0.2 | 0.3 | 0.6 | 0.3 | 0.2 | 0.5 | 0.5 | 0.5 | 0.3 | 0.5 | 0.5 |
| X28 | 0.4 | 0.5 | 0.5 | 0.5 | 0.5 | 0.5 | 0.3 | 0.5 | 0.5 | 0.5 | 0.5 | 0.5 | 0.5 | 0.5 | 0.6 | 0.2 | 0.1 | 0.3 | 0.4 | 0.5 | 0.2 | 0.3 | 0.5 | 0.2 | 0.4 | 0.5 | 0.5 | 0.5 | 0.3 | 0.5 | 0.5 |
| X29 | 0.7 | 0.5 | 0.6 | 0.6 | 0.3 | 0.5 | 0 | 0.6 | 0.6 | 0.5 | 0.6 | 0.6 | 0.6 | 0.5 | 0.7 | 0.5 | 0.2 | 0.2 | 0.5 | 0.5 | 0.5 | 0.3 | 0.5 | 0.5 | 0.4 | 0.5 | 0.7 | 0.7 | 0.5 | 0.8 | 0.8 |
| X30 | 0.5 | 0.5 | 0.5 | 0.5 | 0.4 | 0.6 | 0.3 | 0.5 | 0.5 | 0.5 | 0.5 | 0.5 | 0.5 | 0.5 | 0.5 | 0.2 | 0.1 | 0.1 | 0.4 | 0.5 | 0.3 | 0.3 | 0.2 | 0.3 | 0.2 | 0.5 | 0.5 | 0.5 | 0.2 | 0.5 | 0.5 |
| X31 | 0.4 | 0.5 | 0.6 | 0.6 | 0.4 | 0.5 | 0.4 | 0.5 | 0.6 | 0.5 | 0.5 | 0.5 | 0.5 | 0.5 | 0.5 | 0.1 | 0.3 | 0.1 | 0.3 | 0.5 | 0.3 | 0.3 | 0.2 | 0.2 | 0.3 | 0.5 | 0.5 | 0.5 | 0.2 | 0.5 | 0.5 |
